# Performance of community health insurance in India: findings from empirical studies

**DOI:** 10.1186/1753-6561-6-S1-P9

**Published:** 2012-01-16

**Authors:** Narayanan Devadasan, Bart Criel, Wim Van Damme, Patrick Van der Stuyft

**Affiliations:** 1 Institute of Public Health, Bangalore, India; 2 Department of public health, Institute of Tropical Medicine, Antwerp, Belgium

## Introduction

In India, health care is provided by a mix of government and private providers. While the government health services are ostensibly free, in reality, studies have shown that people have to pay for medicines, diagnostics and other procedures. People approaching the private sector usually end up making out-of-pocket payments (OOP). This has two effects- it can be a substantial and inequitable barrier to accessing health services, and among those who access these services, it can result in impoverishment.

Health insurance is considered as a protective measure against the harmful effects of OOP. Most of the people in India (and especially the poor) are not covered by health insurance. There is a growing movement of community health insurance (CHI) in India, which covers the poorer sections of the Indian community. However, there is little evidence that CHI is able to improve equitable access quality health care and prevent impoverishment. We present the findings of a study on CHI.

## Methods

We made a list of all the CHI programmes in the country and then selected those schemes that provided hospital care. Of these we randomly selected 10 schemes and visited them to document their model. As most of the schemes did not have data on performance, we purposively sampled three of the above 10 schemes (ACCORD, SEWA and KKVS) and conducted a cross sectional survey among the insured and uninsured populations within these schemes.

We interviewed a total of 1400 families and measured variables related to access to hospital care in the past one year, health expenditure among the patients who used hospital care and finally the satisfaction levels. We also collected relevant and available secondary data. Associations were measured using 95% confidence interval as well as multiple regressions.

## Results

From our initial survey, we found that all CHI schemes in the country were organised by non-government organisations (NGO). The schemes can be divided into three broad models – a provider model where the NGO is the organiser, the insurer and the provider of care; an insurer model where the NGO is the organiser and insurer, but purchases care from private hospitals; and finally the agent model where the NGO is just the organiser and purchases insurance from insurance companies and care from private hospitals.

We found that at ACCORD the utilisation of hospital care was 2.2 times higher among insured compared to the uninsured. Insurance was one of the main reasons for this increased access, even after regression.

At ACCORD and SEWA we found that the insurance status was helpful in reducing the OOP payment for the insured. The incidence of catastrophic health expenditure was halved in both the schemes among the insured as compared to the uninsured and this difference was statistically significant. However, in both the schemes, insured patients still had to pay some amount as co-payments.

Patient satisfaction among insured at ACCORD and KKVS were higher in both the schemes but the difference was statistically different only at KKVS.

## Discussion

Our study demonstrates that in India, a well-designed CHI programme is able to increase the access to health care and reduce OOP payments. However, to strengthen these outcomes on health, the schemes need to ensure minimal administrative load for subscribers and patients, increase the benefit package and actively purchase health care from providers.

There are policy implications for the currently introduced Rashtriya Swasthya Bima Yojana (a government-instituted insurance scheme for people below poverty line) and other schemes in terms of organisation of the scheme, the purchasing of care and monitoring and paying attention to the package of services beyond just the financial aspects.

